# Emerging hantavirus infection in wild rodents captured in suburbs of Gwangju Metropolitan City, South Korea

**DOI:** 10.1371/journal.pntd.0010526

**Published:** 2022-06-23

**Authors:** Mi hee Seo, Choon-Mee Kim, Dong-Min Kim, Na Ra Yun, Jung Wook Park, Jae Keun Chung

**Affiliations:** 1 Division of Infectious Disease Investigation, Health and Environment Research Institute of Gwangju City, Gwangju, Republic of Korea; 2 Premedical Science, College of Medicine, Chosun University, Gwangju, Republic of Korea; 3 Departments of Internal Medicine, College of Medicine, Chosun University, Gwangju, Republic of Korea; Chengde Medical University, CHINA

## Abstract

**Background:**

Hemorrhagic fever with renal syndrome (HFRS) caused by hantaviruses is a frequently reported acute hemorrhagic fever in South Korea. These viruses are transmitted by various rodent species such as *Apodemus agrarius*.

**Methodology/Principal findings:**

To investigate hantavirus infection and seroprevalence in rodents, wild rodents were captured from two districts in the suburbs of Gwangju Metropolitan City from January 2016 to December 2018. Nested reverse-transcription polymerase chain reaction (RT-PCR) targeting the hantavirus-specific L segment and indirect immunofluorescence antibody (IFA) assay using Hantaan virus antigen slides were performed. A total of 585 wild rodents were captured—512 *A*. *agrarius*, 49 *Crocidura lasiura*, and 24 *Myodes regulus*. Nested RT-PCR was performed to examine the rate of hantavirus infection in wild rodents, and 1.88% (11/585) of all rodents, 1.17% (6/512) of *A*. *agrarius*, 6.12% (3/49) of *C*. *lasiura*, and 8.33% (2/24) of *M*. *regulus* tested positive. The nucleotide sequence analysis of the eleven PCR-positive products revealed that six PCR products showed over 85% sequence similarity with the Jeju virus, four showed over 99.7% similarity with the Hantaan virus, and one showed over 95.3% homology with the Imjin virus. Moreover, IgG antibodies against the Hantaan virus were detected in 6.15% (36/585) of all rodents, 6.8% (35/512) of *A*. *agrarius*, and 4.17% (1/24) of *M*. *regulus*. IgG antibodies were not detected in *C*. *lasiura*.

**Conclusions/Significance:**

Hantaviruses were detected in all three wild rodent species of *A*. *agrarius*, *C*. *lasiura*, and *M*. *regulus* captured in the suburbs of Gwangju Metropolitan City, South Korea, and it was demonstrated that they were various strains of hantaviruses such as the Hantaan, Jeju, and Imjin viruses.

## Author summary

Hantaviruses (family *Hantaviridae*, genus *Hantavirus*) are rodent viruses that can cause two fatal human diseases—hemorrhagic fever with renal syndrome and hantavirus pulmonary syndrome. These viruses are transmitted by various rodent species such as *Apodemus agrarius*. In the present study, wild rodents captured in the suburbs of Gwangju Metropolitan City were analyzed by nested reverse-transcription polymerase chain reaction targeting the hantavirus-specific L segment and indirect immunofluorescence antibody assay using Hantaan virus antigen slides. A total of 585 wild rodents were captured and 1.88% (11/585) of all rodents, 1.17% (6/512) of *A*. *agrarius*, 6.12% (3/49) of *Crocidura lasiura*, and 8.33% (2/24) of *Myodes regulus* were PCR-positive. Of the eleven PCR-positive wild rodents, six PCR products showed over 85% sequence similarity with the Jeju virus, four showed over 99.7% similarity with the Hantaan virus, and one showed over 95.3% homology with the Imjin virus. Moreover, IgG antibodies against the Hantaan virus were detected in 6.15% (36/585) of all rodents, 6.8% (35/512) of *A*. *agrarius*, and 4.17% (1/24) of *M*. *regulus*. IgG antibodies were not detected in *C*. *lasiura*. When we examined the detection rate of hantavirus genes in different seasons, hantaviruses were most commonly detected in fall (seven cases [5.69%]) and winter (four cases [2.76%]). No hantaviruses were detected in spring or summer. However, the seasonal prevalence of IgG antibodies was higher in spring and summer (12 [7.32%] and 11 [7.10%] cases, respectively) than in fall and winter (seven [5.69%] and six [4.14%] cases, respectively). This study was performed on a monthly basis throughout a three-year period, and thus, it provides reliable data that may provide insight into preventive measures against HFRS. Our results suggest that various types of hantaviruses, including Hantaan, Jeju, and Imjin viruses, are distributed throughout Gwangju Metropolitan City, South Korea. Data regarding the rate of Hantaan virus infection in rodents in Gwangju and knowledge of the hantavirus seroprevalence, species, and genotypes circulating in these domestic rodent species will provide useful information for developing vaccines and diagnostic testing using sequence data. These will help increase preparedness for the emergence of new species.

## Introduction

Hantaviruses (family *Hantaviridae*, genus *Hantavirus*) are rodent viruses that can cause two fatal human diseases—hemorrhagic fever with renal syndrome (HFRS) and hantavirus pulmonary syndrome (HPS) [1,2]. HFRS is prevalent in Asia and Europe, with 150,000 cases reported every year in China, Korea, and Russia [3]. Currently, the HFRS mortality rate is 1–2%, and 300–600 cases have been reported annually in Korea [3]. HFRS is a major rodent-borne infectious disease along with scrub typhus and leptospirosis in Korea and is characterized by fever, headache, stomachache, renal failure, and bleeding. HFRS can be particularly lethal in hypotensive and oliguric phases, as it may induce shock, acute renal failure, acute dyspnea, and bleeding [3]. There are at least 20 serotypes and 36 species known within hantaviruses, and the causative agents of HFRS have been reported as Hantaan, Amur, Seoul, Dobrava, and Puumala viruses. Hantaan virus can cause serious HFRS, whereas Sin Nombre and Andes viruses can induce HPS [2,4,5]. Although Hantaan and Seoul viruses have been detected in Korea, other hantavirus genotypes, including Soochong, Muju, and Imjin viruses, have also been discovered. Among them, Hantaan, Seoul, Soocheong, and Muju viruses are known to be pathogenic in humans [1,4,6]. Transmission of hantaviruses to humans occurs via inhalation of virus-contaminated aerosols or particles from infected rodent excreta (urine, droppings, or saliva) and rarely via rodent bites [3]. Therefore, it is necessary to investigate hantavirus infection in wild rodents to reduce exposure risk and predict future trends in pathogen prevalence and distribution according to seasonal and environmental changes.

This study was conducted to investigate hantavirus infection and seroprevalence in rodents captured in the suburbs of Gwangju Metropolitan City. In addition, we investigated the seasonal hantavirus positivity rate in captured rodents and established the hantavirus genotype via DNA sequencing analysis.

## Methods

### Ethics statement

This study was approved from institutional review board (IRB) of Chosun University. All rodents were euthanized in accordance with an approved animal use protocol from Chosun University Institutional Animal Care and Use Committee (CIACUC) under approval number CIACUC2016-S0003.

### Study site and rodent capture

Wild rodents were captured using Sherman’s live traps in Gwangsan-gu (35°09′19.2′′N, 126°45′05.4′′E) and Buk-gu (35°13′51.7′′N, 126°54′23.8′′E), Gwangju Metropolitan City from January 2016 to December 2018 [7]. The traps were placed at five locations in each district (fallow land, ridges, boundaries between a forest and a field, areas around a cemetery, and waterfront areas) comprising of hills, fields, and agricultural lands. Ten Sherman live traps (3" × 3.5" × 9", USA) were placed at each location to capture rodents. A total of 585 wild rodents were captured and classified by referring to morphological classification keys for wild rodents [8]. Following classification, the rodents were euthanized, and their blood and lung specimens were obtained and stored in a deep freezer (Esco, USA) at -80°C until analysis.

### DNA isolation and PCR amplification

Wild rodent lung tissues stored at -80°C were aseptically homogenized using a cell strainer (70 μm, Falcon, Corning, NY, USA), and viral RNA was extracted using the QIAamp Viral RNA Mini kit (QIAGEN, Hilden, Germany) according to the manufacturer’s instructions. The viral RNA and SuperScript VILO MasterMix (Invitrogen, Massachusetts, USA) were used for hantaviral gene detection and cDNA synthesis. Nested RT-PCR was performed using hantavirus L segment-specific primers, cDNA, and AccuPower PCR PreMix (Bioneer, Daejeon, Korea) [9,10]. The oligonucleotide primers used for nested RT-PCR and PCR product sizes are shown in Table 1. The 2^nd^ (nested) PCR was performed using the same reaction solution as the 1^st^ PCR, with the 1^st^ PCR product used as the template. Positive and negative controls were included in each PCR run. Hantaan virus 76–118 cDNA served as the positive control.

**Table 1:** Oligonucleotide primers used in this study.

Nested RT-PCR targeting hantavirus L-segment	Primers name (Sequence)*	Product size (bp)	References
1^st^ PCR	HAN-L-F1 (5′-ATGTAYGTBAGTGCWGATGC-3′)	450	[9]
	HAN-L-R1 (5′-AA CCADTCWGTYCCRTCATC-3′)		
2^nd^ PCR	HAN-L-F2 (5′-TGCWGATGCHACIAARTGGTC-3′)	380	[9]
	HAN-L-R2 (5′-GCRTCRTCWGARTGRTGDGCAA-3′)		

*Degenerate nucleotide sites are indicated by codes as follows: R = A or G; Y = T or C; W = A or T; B = T or C or G; D = A or T or G; H = A or T or C; I = deoxyinosine.

### Nucleotide sequencing and phylogenetic analysis

For DNA sequencing, the PCR products were purified using a QIAquick Gel Extraction Kit (QIAGEN). The purified DNA fragments were bidirectionally sequenced using PCR primers and an automatic sequencer (ABI Prism 3730XL DNA analyzer, Applied Biosystems, Carlsbad City, CA, USA) at Cosmo Genetech (Daejeon, Korea). The nucleotide sequences were then analyzed using the BLAST network service (Ver 2.33; www.technelysium.com.au/chromas.html) from the National Center for Biotechnology Information (NIH, Bethesda, MD, USA) website. In addition, a phylogenetic tree was constructed based on the partial L segment sequences obtained from the lung tissues of wild rodents with hantavirus infection and those of the L segments of hantaviruses from GenBank using the neighbor-joining method in Clustal X and the Tree Explorer program (DNASTAR, Madison, WI). Bootstrap analysis was conducted using 1000 replicates to increase the confidence level of the phylogenetic tree. LaserGene v6 Program (DNASTAR, Madison, WI) was used for sequence alignment and homology comparison of hantavirus partial L segment sequences obtained from the lung tissues and from GenBank.

### Indirect immunofluorescence antibody (IFA) assay

The IFA assay was performed using Hantaan virus antigen slides to detect hantavirus-specific antibodies [10]. In each well of the slide, 25 μL of wild rodent serum diluted from 1:16 to 1:2,048 was added and reacted with Hantaan virus antigen for 30 min in a humid chamber at 37°C. After washing, the slides were treated with diluted fluorescein isothiocyanate (FITC)-conjugated goat anti-mouse IgG (Sigma, St. Louis, MO, USA) as a secondary antibody. The reacted slides were observed under a fluorescence microscope (Carl Zeiss, Oberkochen, Germany) at 400× magnification after adding a mounting solution (Sigma, St. Louis, Missouri, USA). A titer of 16 was used as the cut-off for screening positive candidates.

## Results

A total of 585 wild rodents were captured in Gwangsan-gu and Buk-gu, Gwangju Metropolitan City from 2016 to 2018. In total, 164 (28.03%), 155 (26.50%), 123 (21.30%), and 143 (24.44%) rodents were captured in spring, summer, fall, and winter, respectively. The species of captured rodents were *Apodemus agrarius* (striped field mouse; 512, 87.52%), *Crocidura lasiura* (big white-toothed shrew; 49, 8.38%), and *Myodes regulus* (royal vole; 24, 4.10%) (Table 2).

**Table 2:** Seasonal distribution of wild rodents captured in Gwangju Metropolitan suburban areas from 2016 to 2018.

Rodents species	Total no. (%)	Spring	Summer	Fall	Winter
		March-May	June-August	September-November	December-February
	585 (100)	164 (28.03)	155 (26.50)	123 (21.03)	143 (24.44)
*Apodemus agrarius*	512 (87.52)	146	154	94	118
*Crocidura lasiura*	49 (8.38)	11	1	26	11
*Myodes regulus*	24 (4.10)	7	0	3	14

Nested RT-PCR targeting the hantavirus L segment was performed using lung specimens from wild rodents. Of the 585 rodents, 11 (1.88%) were PCR-positive for hantaviruses. Viral RNA was detected in six of 512 *A*. *agrarius* (1.17%), three of 49 *C*. *lasiura* (6.12%), and two of 24 *M*. *regulus* (8.33%). The seasonal hantavirus positivity rate determined by using nested RT-PCR was 5.69% (7/123) for rodents captured in fall and 2.76% (4/145) for rodents captured in winter. None of the rodents captured in the summer or spring were PCR-positive.

IFA assay using Hantaan virus antigen slides was performed to investigate the seroprevalence in rodents, and IgG antibodies were detected in 36 of 585 rodents (6.15%), including 35 of 512 *A*. *agrarius* (6.84%) and 1 of 24 *M*. *regulus* (4.17%). The range of IFA IgG titer in the 36 seropositive rodents was 1:16 ~ 1:2,048. No antibodies were detected in *C*. *lasiura* (Table 3). The IgG antibody detection rate in the IFA assay was the highest among the rodents captured in spring (12 of 164 rodents [7.32%]), followed by those captured in summer (7.10%), fall (5.69%), and winter (4.14%) (Table 3). Moreover, 2 of 11 hantavirus PCR-positive rodents were IFA IgG-positive (171110 and 171210). The final IgG antibody titer of the IFA IgG-positive samples was 1:64 and 1:32, respectively (Table 4).

**Table 3:** Prevalence of hantavirus infection in wild rodents from Gwangju Metropolitan suburban areas from January 2016 to December 2018.

		no.	Nested RT-PCR	IFA IgG
			Positive no. (%)	Positive no. (%)
Rodents species				
	Total	585	11 (1.88)	36 (6.15)
	*Apodemus agrarius*	512	6 (1.17)	35 (6.84)
	*Crocidura lasiura*	49	3 (6.12)	0
	*Myodes regulus*	24	2 (8.33)	1 (4.17)
Season				
	Total	585	11 (1.88)	36 (6.15)
	Spring	164	0	12 (7.32)
	Summer	155	0	11 (7.10)
	Fall	123	7 (5.69)	7 (5.69)
	Winter	145	4 (2.76)	6 (4.14)

**Table 4:** Indirect immunofluorescence antibody (IFA) titer and sequencing results of hantavirus PCR positive rodents.

No. of rodents	Rodents species	District	Sequencing results	IFA IgG titer
161006	*Crocidura lasiura*	Buk-gu	Jeju virus	<1:16
161115	*Apodemus agrarius*	Gwangsan-gu	Imjin virus	<1:16
170902	*Apodemus agrarius*	Gwangsan-gu	Hantaan virus	<1:16
170906	*Apodemus agrarius*	Gwangsan-gu	Hantaan virus	<1:16
170910	*Myodes regulus*	Buk-gu	Hantaan virus	<1:16
171110	*Apodemus agrarius*	Gwangsan-gu	Hantaan virus	1:64
171202	*Crocidura lasiura*	Buk-gu	Jeju virus	<1:16
171210	*Apodemus agrarius*	Buk-gu	Jeju virus	1:32
171212	*Apodemus agrarius*	Buk-gu	Jeju virus	<1:16
171213	*Myodes regulus*	Buk-gu	Jeju virus	<1:16
181108	*Crocidura lasiura*	Buk-gu	Jeju virus	<1:16

**Fig 1:**
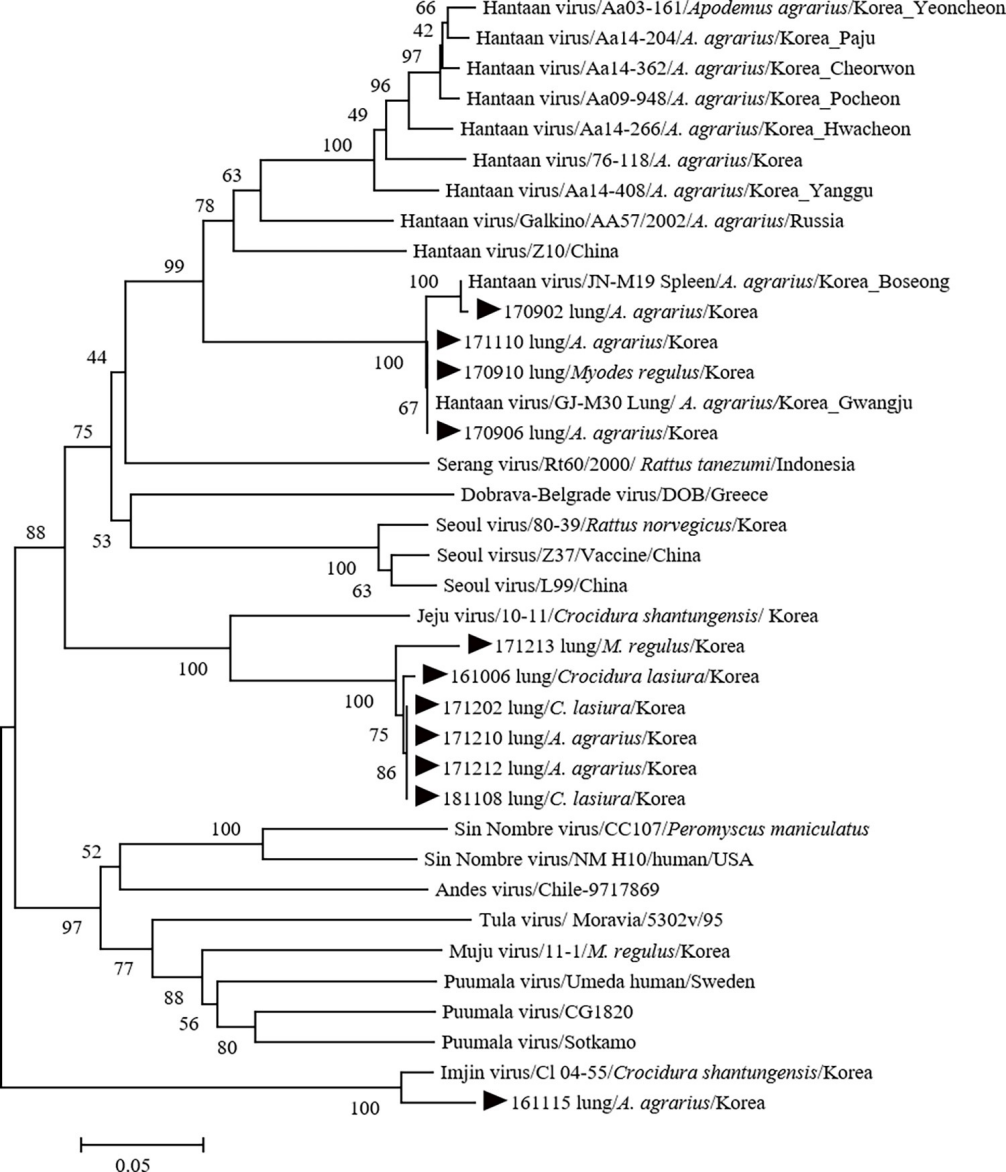
Phylogenetic tree based on the partial L segment sequences (356 bp) from GenBank and Hantavirus-positive wild rodent specimens captured in the Gwangju metropolitan suburban areas from January 2016 to 2018.

A phylogenetic tree was constructed based on the partial nucleotide sequences of the L segment obtained from hantavirus-positive lung specimens and those of the L segments from various hantaviruses available in GenBank as reference sequences (Fig 1). We also compared the similarity of hantavirus L segment nucleotide sequences obtained from GenBank and the 11 hantavirus-positive lung specimens using the LaserGene v6 Program (DNASTAR). Sequence similarity comparison revealed that the L segment nucleotide sequences from six hantavirus-positive lung specimens, 161006, 171202, 171210, 171212, 171213, and 181108, showed 86.1%, 86.1%, 86.0%, 86.0%, 85.6%, and 85.6%, respectively, sequence similarity with the nucleotide sequence of the Jeju virus strain 10–11 (GenBank accession number HQ834697), detected in *Crocidura shantungensis* captured in South Korea in 2010, but formed a cluster different from that of the Jeju virus in the phylogenetic tree. The L segment nucleotide sequences from three hantavirus-positive lung specimens (170906, 170910, and 171110) showed 100% sequence similarity with the Hantaan orthohantavirus isolate GJ-M30 (GenBank accession number MN507674.1), detected in *A*. *agrarius* in South Korea, and formed a cluster with Hantaan virus in the phylogenetic tree. The L segment nucleotide sequence from one hantavirus-positive lung specimen, 170902, showed 99.7% sequence similarity with the Hantaan orthohantavirus isolate JN-M19 (GenBank accession number MN507690.1), detected in *A*. *agrarius* in South Korea, and formed a cluster with Hantaan virus in the phylogenetic tree. The L segment nucleotide sequences of the remaining hantavirus-positive lung specimen, 161115 showed 95.3% sequence similarity with the Imjin virus strain Cl 04–55 (GenBank accession number EF641807.1), detected in *C*. *lasiura* captured in South Korea, and formed a cluster with the Imjin virus in the phylogenetic tree. Of the four hantaviruses that showed the highest homology with Hantaan virus, three (170902, 170906, and 171110) were detected in *A*. *agrarius* and one (170910) in *M*. *regulus*. Of the six hantaviruses that showed the highest homology with Jeju virus, three (161006, 171202, and 181108) were detected in *C*. *lasiura*, two (171210 and 171212) in *A*. *agrarius*, and one (171213) in *M*. *regulus*. One hantavirus with the highest homology with the Imjin virus (161115) was found in *A*. *agrarius*.

## Discussion

HFRS is a major rodent-borne infectious disease affecting at least 500 individuals in South Korea every year (http://www.kdca.go.kr/npt/biz/npp/ist/simple/simplePdStatsMain.do

). Extra caution is warranted to prevent HFRS during the fall season. In particular, the seasonal prevalence of HFRS, which mainly affects people who work outdoors, can be attributed to increased activities in rural areas, such as harvesting, and increased rodent activities that occur in autumn [11]. It is necessary to understand the epidemiologic characteristics and genotypes of hantaviruses, the causative agents of HFRS, to establish preventive measures in humans.

In this study, we performed nested RT-PCR targeting the hantavirus L segment and found three hantaviruses, namely, the Jeju, Hantaan, and Imjin viruses, in wild rodents. In nested RT-PCR performed to examine hantavirus genotypes, 11 of 585 samples (1.88%) were PCR-positive, and hantavirus genes were detected in all the three species—*A*. *agrarius*, *C*. *lasiura*, and *M*. *regulus*. We examined the detection rate of hantavirus genes in different seasons; hantaviruses were most commonly detected in fall (seven cases [5.69%]) and winter (four cases [2.76%]). No hantaviruses were detected in spring or summer.

When the IgG cut-off value (≥1:16) was used in the IFA, 36 of 585 serum samples (6.15%) tested positive for IgG antibodies against hantaviruses. Two of 36 IFA IgG-positive rodents were PCR-positive for hantaviruses. In particular, the seasonal prevalence of IgG antibodies was higher in spring and summer (12 [7.32%] and 11 [7.10%] cases, respectively) than in fall and winter (seven [5.69%] and six [4.14%] cases, respectively). The antibody positivity rate identified in this study was slightly lower than or similar to the rates reported by Song et al. and Nam et al. (13.4% and 7.9%, respectively) [12,13].

According to Lim et al., the positivity rate of Hantaan virus IgG antibodies in wild rodents in South Korea was very high at 25.8% (162/629), which was demonstrated using the IFA assay. When examining the IgG antibodies as per seasons, the positivity rate was higher in fall (29.6% [75/253]) than in spring (23.1% [87/376]). When examining the IgG antibodies by rodent species, *A*. *agrarius* showed the highest positivity rate of 26.9% (152/561), followed by *A*. *peninsulae* (21.4% [3/14]) and *M*. *regulus* (17.2% [5/29]) [14].

In 2011, Ryou et al. performed PCR targeting the S segment of the Hantaan virus in wild rodents captured from five provinces in South Korea and reported a PCR-positivity rate of 3.3% and varying positivity rates of Hantaan virus antibodies from 4–29% in different regions. In addition, they reported high antibody prevalence between March, May, October, and December and that the seasonal pattern of Hantaan virus positivity in rodents affected the seasonal prevalence of HFRS in humans [15].

Of the 11 hantaviruses detected in rodents in this study, six showed over 85% sequence similarity with the Jeju virus, four showed over 99.7% sequence similarity with Hantaan virus, and one showed 95.3% sequence similarity with the Imjin virus. Of the hantaviruses that were homologous to the Jeju virus, three were detected in *C*. *lasiura*, two in *A*. *agrarius*, and one in *M*. *regulus*. Of the hantaviruses that showed homology with Hantaan virus, three were detected in *A*. *agrarius* and one in *M*. *regulus*. One hantavirus that showed homology with the Imjin virus was detected in *A*. *agrarius*.

In 2009, Song et al. reported Imjin virus, a novel hantavirus, in the lungs of *C*. *lasiura* (Ussuri white-toothed shrew) that had been captured in the demilitarized zone of South Korea. Seven cases of Imjin virus were confirmed by RT-PCR out of a total of 115 *C*. *lasiura* individuals captured. The prevalence of Imjin virus was highest in the fall season based on the IFA assay and RT-PCR test results [16]. In addition, 12 cases of Imjin virus were confirmed by RT-PCR among the 466 *C*. *lasiura* individuals captured in the Republic of Korea between 2004–2010 [17]. No viruses other than Imjin were detected in the captured *C*. *lasiura*, so the major animal host of Imjin virus was reported as *C*. *lasiura*.

In 2012, Arai et al. detected a novel hantavirus, Jeju virus, in Asian lesser white-toothed shrews (*Crocidura shantungensis*) that were captured on Jeju Island, South Korea. Eight cases of Jeju virus were confirmed by RT-PCR among the 51 *C*. *shantungensis* captured on Jeju Island. But the virus was not found in the 28 *C*. *shantungensis* individuals that had been captured on mainland Korea. Moreover, there is a divergent ancestral lineage between the Imjin and Jeju viruses despite the close phylogenetic relationship between the two reservoir hosts [18].

Hantaviruses detected in rodents captured in South Korea include Hantaan virus in striped field mice (*Apodemus agrarius*), Seoul virus in brown rat (*Rattus norvegicus*), Soochong virus in Korean field mice (*Apodemus peninsulae*), and Muju virus in royal voles (*Myodes regulus*) [18]. The results of this study show that *A*. *agrarius*, *C*. *lasiura*, and *M*. *regulus* may transmit hantaviruses to humans.

The high PCR positivity rates of hantavirus in wild rodents captured in fall and winter somewhat closely match the seasonal detection rates reported by Park, who analyzed 6,132 HFRS cases recorded by the Korea Centers for Disease Control from 2002 to 2016 at a national scale [19]. He reported that the quarterly incidence of HFRS in humans was statistically higher in the third (12.9%) and fourth (67.5%) quarters.

The results of this study are meaningful in that at least three hantavirus strains, namely Hantaan, Jeju, and Imjin viruses, were detected using nested RT-PCR targeting the hantavirus L segment and that the prevalence rate for Hantaan virus-specific IgG antibodies was measured in rodents captured within a limited area, namely, Gwangju Metropolitan City, South Korea. Above all, the detection of Jeju virus in three species—*C*. *lasiura*, *A*. *agrarius*, and *M*. *regulus—*other than the previously reported *C*. *shantungensis* host suggests high infection prevalence and frequency of spillover events. This study is the first to present such findings. The same goes for the detection of Imjin virus in *A*. *agrarius*. Unlike previous studies that were conducted during a specific period of the year, this study was performed on a monthly basis throughout a three-year period, and thus, it provides reliable data that may provide insight into preventive measures against HFRS. The rate of Hantaan virus infection in rodents in Gwangju and the information about various hantavirus strains presented in this study will be useful for the development of vaccine candidates and preventive measures for HFRS.

In conclusion, potentially pathogenic hantaviruses were detected in all three rodent species that transmit HFRS in Gwangju Metropolitan City. Various types of hantaviruses, including Hantaan, Jeju, and Imjin viruses, are distributed throughout Gwangju. Knowledge of the hantavirus seroprevalence/ species/genotypes circulating in these domestic rodent species will provide an useful information for the rapid diagnosis of human infections as well as the potential for emergence of new species.
